# Biomass production and identification of suitable harvesting technique for *Chlorella* sp. MJ 11/11 and *Synechocystis* PCC 6803

**DOI:** 10.1007/s13205-015-0360-z

**Published:** 2016-01-27

**Authors:** Amrit Lal, Debabrata Das

**Affiliations:** Department of Biotechnology, Indian Institute of Technology Kharagpur, Kharagpur, 721302 West Bengal India

**Keywords:** Harvesting, Flocculation efficiency, Chitosan, Electrolytic coagulation-floatation, *Chlorella* sp. MJ 11/11

## Abstract

Microalgae that can grow fast and convert solar energy into chemical energy efficiently are being considered as a promising feedstock of renewable biofuel. Mass production of microalgal oil faces a number of technical barriers that make the current production of biodiesel economically unfeasible. Small size (≈1–20 μm) and negatively charged surface of the microalgal cells pose difficulties in the process of harvesting. This leads to significant increase in the overall cost of biomass production. The present study explored different methods and conditions for harvesting of *Chlorella* sp. MJ 11/11 and *Synechocystis* PCC 6803. A customized air-lift reactor was used for the cultivation of biomass under photoautotrophic condition. Significant improvement in the rate of productivity of biomass was observed. Maximum biomass productivity of 0.25, 0.14 g L^−1^ d^−1^ for *Chlorella* sp. MJ 11/11 and *Synechocystis* PCC 6803, respectively, were obtained. Various flocculation techniques viz. auto-flocculation, inorganic, chitosan and electrolytic flocculation were used for the recovery of biomass. Among all the techniques, electro-flocculation showed high flocculation efficiency (98 %) and floatation of floc causing easy harvesting. Moreover, low-cost and easy control of the process justify electro-flocculation as a most suitable and promising technique for the recovery of microalgal cells.

## Introduction

Microalgal biomass is considered as a renewable feedstock for production of fuels, feed, nutraceutical and pharmaceutical products. Algal cultivation requires minimum nutritional input. They may be used for wastewater bioremediation. The biomass generated from such processes can be used for production of third generation of biofuels like biodiesel, bioethanol, biohydrogen, biobutanol, and other biofuels (Skjanes et al. 2007; Nayak et al. 2013). For the commercialization of bioproducts from microalgae, a large amount of algal biomass is required. Harvesting plays a significant role in product development which separates the microalgal biomass from their liquid media. Development of cost-effective and environment-friendly methods for harvesting of algal biomass are still challenging for the future. Separation of algae is difficult mainly due to smaller particle size (1–20 μm), low cell density and low specific gravity of algal (Ghernaout and Ghernaout 2012). Several species of microalgae have different characteristics such as size, shape, and motility. These can influence their behavior towards flocculation techniques unlike other non-living particles in suspension (Vlaski et al. 1997). Most of the commercial organizations use centrifugation, the traditional method of harvesting. But it is an energy intensive process since it consumes a good amount of electric power (Chen et al. 2011). Some microalgae can be harvested using filtration, but membrane fouling limits the process due to clogging of the pore by extracellular organic matter (Wu et al. 2012). It can also be harvested using foam fractionation, but the energy consumption for large-scale harvesting systems is high. If algae oil is to be a viable future energy source, the harvesting problem will need to be overcome. So, development of an efficient harvesting strategy is a major challenge in the commercialization of products from microalgae. This step accounts for 20–30 % of the total cost of production (Grima et al. 2003). A suitable approach to harvesting is needed to minimize the cost, energy consumption and applicable for a wider range of conditions.

The harvesting of microalgae by flocculating agents has been found more promising over other methods. It allows to flocculate a large quantity of microalgal cultures and applies to a broad range of species (Uduman et al. 2010). The lower energy consumption and costs render it as an attractive technique. Microalgal cell suspensions are stabilized by the surface charge of the cells. Microalgal cells carry a negative charge that prevents aggregation of cells in suspension (Nishifi et al. 2011). Various flocculation methods are used to destabilize the cell suspension. The addition of flocculants destabilizes the cell suspension for the creation of aggregates of microalgal cells. The surface charge can be neutralized or reduced by adding flocculants such as multivalent cations and cationic polymers to the broth. The increase in effective particle size of the cell leads to sedimentation and hence microalgal biomass can be recovered. Moreover, an inexpensive and nontoxic flocculation method is desired which would be effective in low concentration (Milledge and Heaven 2013). The flocculation efficiency varies with different flocculating agents, microalgal species and culture conditions (Henderson et al. 2008). The flocculation efficiency mainly depends on an electric neutralization effect after hydrolysis of the coagulant and emerging compression of the electrical double layer. This leads to the formation of the adsorbent bridges resulting in a macromolecular complex.

The aim of the present study was to increase biomass production and identification of suitable method for harvesting of *Chlorella* sp. MJ 11/11 (lipid rich) and *Synechocystis* PCC 6803. Different flocculating methods such as inorganic flocculants alum and FeCl_3_, bio-flocculant like chitosan, auto-flocculation, and electrolytic coagulation-flocculation were evaluated to recover the microalgal biomass from diluted suspension.

## Materials and methods

### Cultivation of microalgae

Microalgae species namely, *Chlorella* sp. MJ 11/11 and *Synechocystis* PCC 6803 were obtained from the culture collection of algae at IARI, New Delhi, India and Uppsala University, Sweden, respectively. *Chlorella* sp. MJ 11/11 and *Synechocystis* PCC 6803 were grown in a customized airlift reactor (1.4 L) under 120 µmol m^−2^ s^−1^ light intensity. The light intensity was measured using a quantum sensor (LI-COR, Model LI-190SA, NE, USA) and the light meter (LI-COR, Model LI-250A, NE, USA). TAP minus acetate medium was selected as the growth medium for *Chlorella* sp. MJ 11/11 and BG11 for *Synechocystis* PCC 6803. The composition of TAP minus acetate media contained 2.42 g L^−1^ Tris base, 25 mL L^−1^ TAP salt stock solution (15.0 g L^−1^ NH_4_Cl, 4.0 g L^−1^ MgSO_4_·7H_2_O, 2.0 g L^−1^ CaCl_2_·2H_2_O, 0.375 ml L^−1^ PO_4_ stock solution (28.8 g per 100 mL K_2_HPO_4_, 14.4 g per 100 mL KH_2_PO_4_), 1 ml L^−1^ Hutner trace metals (21.6 g per 100 mL H_2_O EDTA: Titriplex II, 11 g per 50 mL H_2_O ZnSO_4_·7H_2_O, 5.7 g per 100 mL H_2_O H_3_BO_3_, 2.53 g per 25 mL H_2_O MnCl_2_ × 4H_2_O, 0.805 g per 25 mL H_2_O CoCl_2_·6H_2_O,0.785 g per 25 mL H_2_O CuSO_4_·5H_2_O, 0.55 g per 25 mL H_2_O (NH_4_)_6_Mo_7_O_24_·4H_2_O, 2.495 g per 25 mL H_2_O FeSO_4_·7H_2_O), 1 ml L^−1^ vitamins stock solution (0.5 mg L^−1^ cyanocobalamin (B_12_), 100 mg L^−1^ thiamine HCl, 0.5 mg L^−1^ Biotin), glacial acetic acid was absent in TAP [-acetate] medium. Initial pH of the medium was set at 7.2. 1 L of BG-11 medium contained 0.04 g of K_2_HPO_4_, 0.075 g of MgSO_4_·7H_2_O, 0.036 g of CaCl_2_·2H_2_O, 6.0 mg of citric acid, 6.0 mg of ferric ammonium citrate, 1.0 mg of Na_2_EDTA, 0.02 g of Na_2_CO_3_ and 1.0 mL of trace metal solution A5. 1 L of the trace metal solution A5 contained 2.86 g of H_3_BO_3_, 1.81 g MnCl_2_·4H_2_O, 0.22 g ZnSO_4_·7H_2_O, 0.39 g of Na_2_MoO_4_·2H_2_O, 0.079 g of CuSO_4_·5H_2_O and 49.4 mg of Co (NO_3_)_2_·6H_2_O. The biomass harvesting experiments were carried out at the stationary phase of growth at room temperature.

### Air-lift photobioreactor

**Fig. 1:**
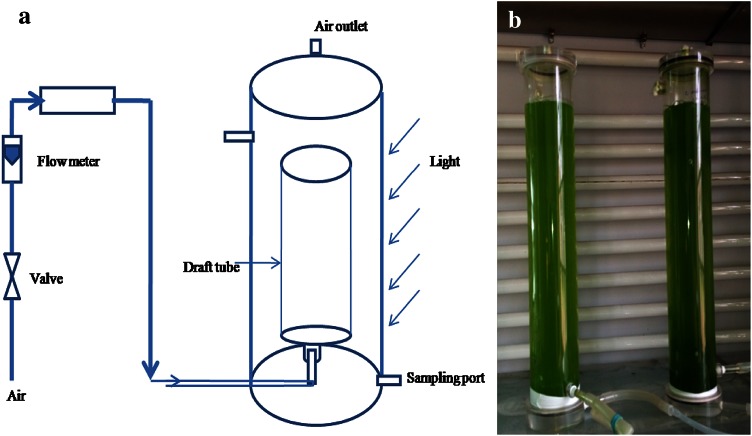
**a** Schematic diagram of an air-lift reactor, **b** experimental setup of air-lift reactor

Air-lift photobioreactor was made up of plexiglass of thickness 5 mm. Air-lift bioreactors were of dimensions diameter 7 × 42.3 cm with two sides opening near the top and bottom of the reactor. The diameter of the draft tube of an air-lift reactor was 3 cm. The surface by volume (S/V) ratio and the *A*_d_ */A*_r_ were 0.57 cm^−1^ and 4.4, respectively where, *A*_d_ and *A*_r_ are the area of downcomer and riser, respectively. Airstream was provided by a sparger attached at the bottom (Fig. 1a, b). Photobioreactors were constantly illuminated by tube light at a light intensity of 120 μmol m^−2^ s^−1^.

### Determination of cell concentration

The biomass concentrations (dry cell weight, g/L) of microalgae were calculated from measurements of the optical density (OD) of cultures at 750 nm according to the following equations: *Synechocystis* PCC 6803, *y* = 0.499*x* + 0.125, *R* ^2^ = 0.967, *Chlorella* sp. MJ 11/11: *y* = 0.420*x* + 0.166, *R* ^2^ = 0.954. To obtain the same concentration of *Chlorella* sp. MJ 11/11 and *Synechocystis* PCC 6803 broth culture was concentrated by centrifugation (Eppendorf, Hamburg) at 4000 rpm for 5 min and diluted by their respective supernatant according to need and equalized the OD at 750 nm. The pH values were monitored using a desktop pH meter (Orion 5 stars, Thermo Scientific).

### Determination of volumetric biomass productivity

Volumetric biomass productivity was calculated by following equation.

$${\text{Biomass}}\,{\text{productivity}}\,\left( {{\text{gL}}^{{\text{ - } 1}} {\text{d}}^{{\text{ - } 1}} }\, \right){ \,=\, }\frac{{\left( {X_{ 2} \text{ - }X_{ 1} } \right)}}{{\left( {t_{ 2} \text{ - }t_{ 1} } \right)}}$$

where, *X*_1_ and *X*_2_ are biomass concentrations (g/L) at time *t*_1_ and *t*_2_.

### Determination of flocculation efficiency

The optical density of the supernatant was measured from the middle of the height of the clarified layer (Nova Spectro, Amersham pharmacia biotech). The flocculation efficiency was calculated according to equation.

$${\text{Flocculation}}\,{\text{efficiency}}\,{ = }\,\left( {{\text{OD}}_{\text{i}} - {\text{ OD}}_{\text{f}} / {\text{OD}}_{\text{i}} } \right) \times 1 0 0$$

where OD_i_ = Initial OD and OD_f_ = final OD at 750 nm.

### Stock preparation of flocculants

200 mg of ferric chloride, alum and chitosan were weighed (Sartorius) individually and dissolved in 20 ml of distilled water to obtain 10 mg/ml of individual stock. Chitosan (Sigma-Aldrich) solution was made in 1 % acetic acid solution according to Divakaran and Pillai (2002).

### Experimental setup for chemical flocculation

Auto-flocculation of *Chlorella* MJ 11/11 was studied by taking a pH range of 8–12.5. The pH of the TAP minus acetate medium was adjusted by adding 1 M NaOH and 1 M HCl. Various concentration of ferric chloride (100–400 mg/L), potassium aluminum sulfate (100–500 mg/L), chitosan (5–25 mg/L) solution were added and mixed properly. It was kept undisturbed for settling. The Supernatant was collected from the middle of beaker and OD was taken at 750 nm and flocculation efficiency was determined by the above formula. The experiment was done with 20 mL culture in 25 mL beaker at room temperature.

### Electrolytic flocculation (ECF)

The electrolytic cell with a diameter of 10 cm, a height of 15 cm was a glass reactor equipped with a magnetic stirrer. Electrodes plates (5.5 × 4 cm) made from stainless steel were used as an electrode. The electrodes were submerged into the algal solution and were connected to a direct current power supply source. Inter electrode distance of 4 cm was maintained throughout the experiments. In 1 L beaker 400 ml of *Chlorella* sp. MJ 11/11 and *Synechocystis* PCC 6803 culture was used for the experiment. It was operated at different DC voltage (6, 9 and 12 V). During ECF, the microalgal suspension was stirred using magnetic stirrer at 150 rpm. Agitation was stopped with the initiation of the microalgal floc formation. Different concentration of NaCl (0.5 and 1 g L^−1^) was used to make the culture broth saline. Sample collection was done at every 15 min interval to find out the flocculation efficiency.

### Zeta potential measurement

The zeta potential of the algae species was determined by Zetatrac analyzer using three mL cell dispersions in their respective medium before and after flocculation at various pH in the auto-flocculation study.

### Cost estimation of different flocculation method

Flocculation cost of microalgal biomass following different methods was estimated with flocculation efficiency ≥90 %. Chemicals were obtained from different vendor alum, ferric chloride from SRL and chitosan from Sigma–Aldrich. Price may vary from supplier to supplier. Power consumption was calculated in the process of electro-flocculation according to Vandamme et al. (2011). Electro-flocculation cost was determined on the basis of per unit power consumption when per unit price of electricity is 7.22 Rs/KWH (West Bengal, India).

## Results and discussion

### Biomass production of microalgae in air-lift bioreactor

**Fig. 2:**
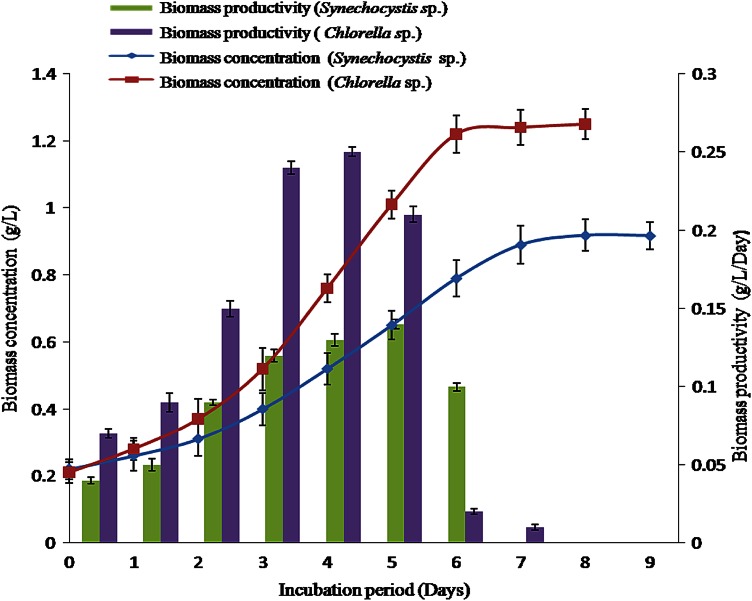
Biomass concentration and biomass productivity of the *Chlorella* sp. MJ 11/11 and *Synechocystis* PCC 6803 in air-lift bioreactor

Growth potential of *Chlorella* sp. MJ 11/11 and *Synechocystis* PCC 6803 was evaluated under the phototrophic indoor condition in customized air-lift reactor. After 9 d of cultivation, the maximum concentration of biomass 1.25 g/L and maximum productivity 0.25 gL^−1^d^−1^ for *Chlorella* sp. MJ 11/11 were obtained. *Synechocystis* PCC 6803 produced maximum biomass 0.918 g L^−1^ and maximum productivity was 0.14 g L^−1^ d^−1^ (Fig. 2). Comparative analysis on the biomass productivities is shown in Table 1 which justifies the suitability of the present process for algal biomass production. *Chlorella* sp. MJ11/11 is a fast growing microalga as compared to some other microalgae spp. So, it was considered in the present study. The air-lift reactor was found to be a suitable photobioreactor for enhancing the biomass productivity. The air-lift reactor has the advantage of creating circular mixing where liquid culture passes continuously through dark and light phase that provide flashing light effect to algal cells (Barbosa et al. 2005). The moving cultivation surface helps in increasing the biomass productivity. These results indicated that *Chlorella* sp. MJ 11/11 could be a promising feedstock for biodiesel production in the air-lift reactor.

**Table 1:** Comparison of biomass productivity of different oleaginous microalgae with *Chlorella* sp. MJ 11/11 and *Synechocystis* PCC 6803

Oleaginous microalgae	Conditions	Biomass productivity (g/L/day)	References
*Chlorella emersonii*	Tubular photo-bioreactor, Watanabe’s medium, air phototrophic	0.04	Scragg et al. (2002)
*Chlorella vulgaris* INETI 58 *Nannochloropsis* sp.	Air-lift bioreactors then in polyethylene bags with bubbling air, phototrophic	0.18 0.09	Gouveia and Oliveira (2009)
*Scenedesmus obliquus*	Erlenmeyer flasks, N 11 medium, air, phototrophic	0.06	Mandal and Mallick (2009)
*Chlorella vulgaris*	Bioreactor, BG-11 medium, 10 % CO_2_, phototrophic	0.105	Yoo et al. (2010)
*Chlorella* sp. MJ 11/11* *Synechocystis* PCC 6803**	Air-lift reactor, TAP* and BG-11**, air, phototrophic	0.25* 0.14**	Present study

* Biomass productivity of Chlorella sp. MJ 11/11 using TAP medium is 0.25 g/L/day and that of Synechocystis PCC 6803 using BG-11 medium is 0.14 g/L/day

### Auto-flocculation in *Chlorella* sp. MJ 11/11 and *Synechocystis* PCC6803

**Fig. 3:**
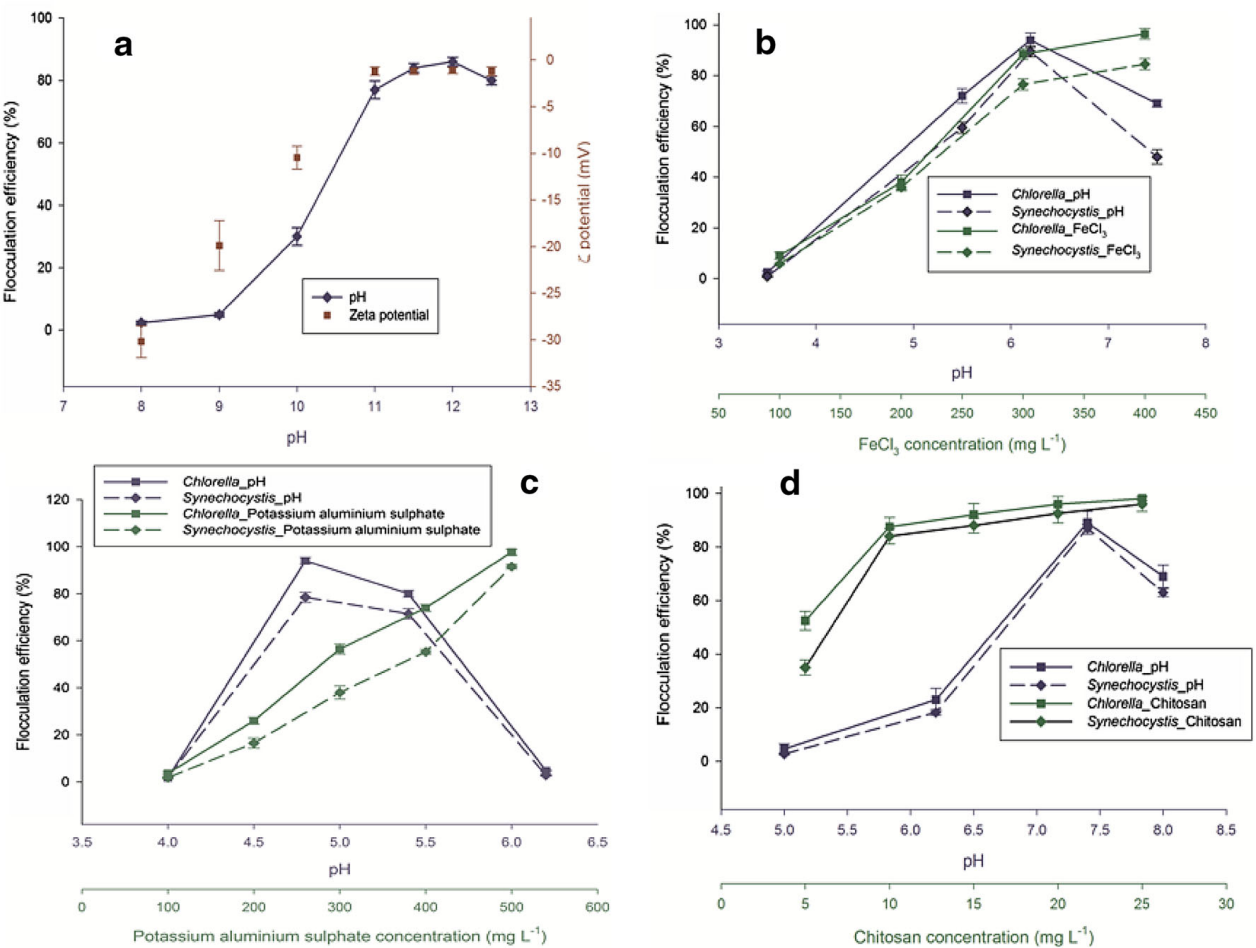
**a** Auto-flocculation efficiency and ζ potentials of *Chlorella* sp. MJ 11/11 at different pH; **b** flocculation efficiency of *Chlorella* sp. MJ 11/11 and *Synechocystis* PCC 6803 at different dosage and pH of FeCl_3_, **c** potassium aluminum sulfate, and **d** chitosan

Auto-flocculation was studied in *Chlorella* MJ11/11 and *Synechocystis* PCC 6803 by increasing the pH (8–12.5). High flocculation efficiency was observed in case of *Chlorella* sp. MJ 11/11 (Fig. 3a). Flocculation efficiency increased sharply after pH 11 and was found maximum (85 %) at pH 12. At this pH, the zeta potential was found minimum. When the zeta potential is close to zero, particles approach each other mainly due to Van der Waals forces. When this happens, particles aggregate and flocculation occurs (Vandamme et al. 2013). It has been reported that above pH 10, calcium and magnesium ions present in the culture broth are precipitated as magnesium and calcium hydroxide. These are positively charged ions and adsorbed at negatively charged microalgal cells surface. Hence, neutralizes the negative charge and helps in reducing the repulsion forces among microalgal cells that lead to floc formation (Uduman et al. 2010). In case of *Synechocystis* PCC 6803 grown in BG-11 medium, no auto-flocculation was observed. It is suggested that proper concentrations of calcium and orthophosphate ions in the medium are important for auto-flocculation (Sukenik and Shelef 1984). Probably, hydroxides of calcium and magnesium formed in BG-11 were not sufficient to neutralize the negative charge present on its cell surfaces. The pH threshold is strain specific, e.g. *Scenedesmus dimporphus* only flocculates above pH 8.5 (Sukenik and Shelef 1984) while pH 12 is required for *Chlorella* sp. flocculation. These observations suggested that auto-flocculation depends on both the medium and the microorganism (Yahi et al. 1994).

Auto-flocculation was found biomass concentration dependent (Table 2). At high biomass concentration (2.8 g/L) 32 % flocculation was observed while 0.98 g/L improved the flocculation efficiency to 82 %. Studies on *Chlorella vulgaris* (García-Pérez et al. 2014) suggested that the amount of magnesium hydroxide required for flocculation increases with increasing biomass concentration. Since the concentration of magnesium ions are constant in media which leads to less concentration of magnesium and calcium hydroxide formation hence less flocculation efficiency was found at higher biomass concentration.

**Table 2:** Effect of different biomass concentrations on the flocculation efficiency of *Chlorella* sp MJ 11/11 and *Synechocystis* PCC 6803

Flocculation techniques	Biomass concentration (g L^−1^)	Flocculation efficiency (%)	
		*Chlorella* sp. MJ 11/11	*Synechocystis* PCC 6803
Auto flocculation	0.98	82.0 ± 1.46	–
	1.50	62.0 ± 2.19	–
	2.80	32.0 ± 2.32	–
Chemical flocculation (FeCl_3_)	1.00	98.0 ± 1.12	87.0 ± 1.71
	1.70	92.0 ± 1.78	82.0 ± 1.34
	2.60	82.0 ± 2.17	74.0 ± 2.12
Chemical flocculation (Alum)	1.00	98.0 ± 0.98	93.0 ± 1.34
	1.70	92.0 ± 1.47	90.0 ± 2.13
	2.80	87.0 ± 2.35	82.0 ± 2.06
Bioflocculation (Chitosan)	1.00	98.0 ± 1.16	88.0 ± 2.17
	2.20	86.0 ± 2.34	85.0 ± 1.49
	3.20	82.0 ± 1.78	82.0 ± 1.77
Electro-flocculation	0.98	98.0 ± 1.13	93.4 ± 1.67
	1.20	87.0 ± 1.89	72.9 ± 2.56
	1.60	54.0 ± 2.18	50.4 ± 2.84

### Inorganic cation-based flocculation

Aluminum and iron salts have been used as coagulants in wastewater treatment. These flocculants have been found effective in removing the impurities, colloidal particles and dissolved organic substances from water bodies (Duan and Gregory 2003). These inorganic coagulants have been reported for various algae biomass separation (Wyatt et al. 2012). In the present study, the flocculation efficiency was found proportional to the coagulant concentration (Fig. 3b). Maximum flocculation efficiencies 98 and 85 % were observed in the case of *Chlorella* sp. MJ 11/11 and *Synechocystis* PCC 6803, respectively at 400 mg/L of ferric chloride. In the case of potassium aluminum sulfate, maximum efficiency (98.6 %) for *Chlorella* sp. MJ 11/11 and 92.3 % for *Synechocystis* PCC 6803 were observed at a concentration of 500 mg/L. The pH was found suitable between 4.8 and 5.4 (Fig. 3c). The effectiveness declined beyond the above pH range. At low biomass concentrations, higher flocculation efficiencies were observed. It was 98 and 87 % for *Chlorella* sp. MJ 11/11 and *Synechocystis* PCC 6803, respectively, at 1 g/L biomass concentration for ferric chloride and a similar type of result was found for potassium aluminum sulfate. The decrement of 10 % was found in the flocculation efficiency from low to higher biomass concentration for both species (Table 2). Mechanism of flocculation of potassium aluminum sulfate and ferric chloride was found to be similar as they form hydroxide when dissolving in water. It was suggested that aggregation of the cells occur due to the interaction between the cationic hydroxide complexes and anionic algal cell surface. Therefore, surface neutralization occurred in accordance with the principle of adsorption coagulation. The lower flocculant concentration follows charge neutralization mechanism resulting less biomass separation. While, at higher dose there is an extensive formation of hydroxide precipitate that attributes to sweep flocculation where mesh like algal floc patch synthesis entrapped free cells causing elevated efficiency (Duan and Gregory 2003). Algal cell surface carries a variety of functional groups whose characteristics changes depending upon pH (Wu et al. 2012). The addition of ferric chloride in the medium results in decrease in the pH of the solution due to the formation of ferric hydroxide and hydrochloric acid (Wyatt et al. 2012). Therefore, for better flocculation an optimum pH is necessary.

### Chitosan-mediated flocculation

Chitosan is non-toxic, biodegradable and linear cationic polymer (Chen et al. 2011). In acidic conditions, chitosan has positively charged amine groups that are adsorbed to the negatively charged colloidal particle surface (Roussy et al. 2005). Flocculation occurs predominantly by the inter-particle bridging via polymer molecules that adsorb in a loop and tail conformation.

Flocculation studies over a range of chitosan concentration (10–25 mg/L) were conducted with *Chlorella* sp. MJ 11/11 and *Synechocystis* PCC 6803 (Fig. 3d). Initial biomass concentration was kept constant at 0.98 g/L. Flocculation efficiency of 86–90 % was observed with 10 mg/L chitosan concentration at pH 7.4. With increasing flocculants dose beyond 10 mg/L, over 90 % flocculation efficiency was achieved within 5–6 min resulting in the final efficiency of 98 %. At higher concentration of chitosan (25 mg/L) in both organisms, similar flocculation efficiency (98–98.5 %) was observed. Some have reported maximum flocculation at pH 7 (Divakaran and Pillai 2002) whereas pH 8.5 for *Chlorella sorokiniana* (Chen et al. 2011; Grima et al. 2003). However, in the present study pH 7.4 was found to be optimum for flocculation that was 94 and 90 % for *Chlorella* sp. MJ 11/11 and *Synechocystis* PCC 6803, respectively. Roussy et al. (2005) identified that at intermediate pH both the coagulation and flocculation processes occurred in biopolymer mediated flocculation of bentonite suspensions. Further, the difference in optimal pH and flocculation efficiency of microalgae might be due to the difference in culture media, growth conditions and unique strain properties, such as cell morphology, extracellular organic matter and cell surface charge (Xu et al. 2013). The flocculation efficiency showed a decreasing trend with the rise in biomass concentration (Table 2). Since, the increase in biomass concentration increases total negative surface charges in the solution. Hence, a more positive charge would be required for neutralization. Therefore, at constant flocculant concentration decreasing trends with biomass was found.

### Electrolytic coagulation-flocculation

The electrolytic flocculation experiments are based on the principle of the movement of electrically charged particles in an electric field. Microalgae have a negative surface charge that causes them to be attracted towards the anode during the electrolysis of the algal suspension. They reach to the anode and lose their charges that make them able to form algal aggregates.

**Fig. 4:**
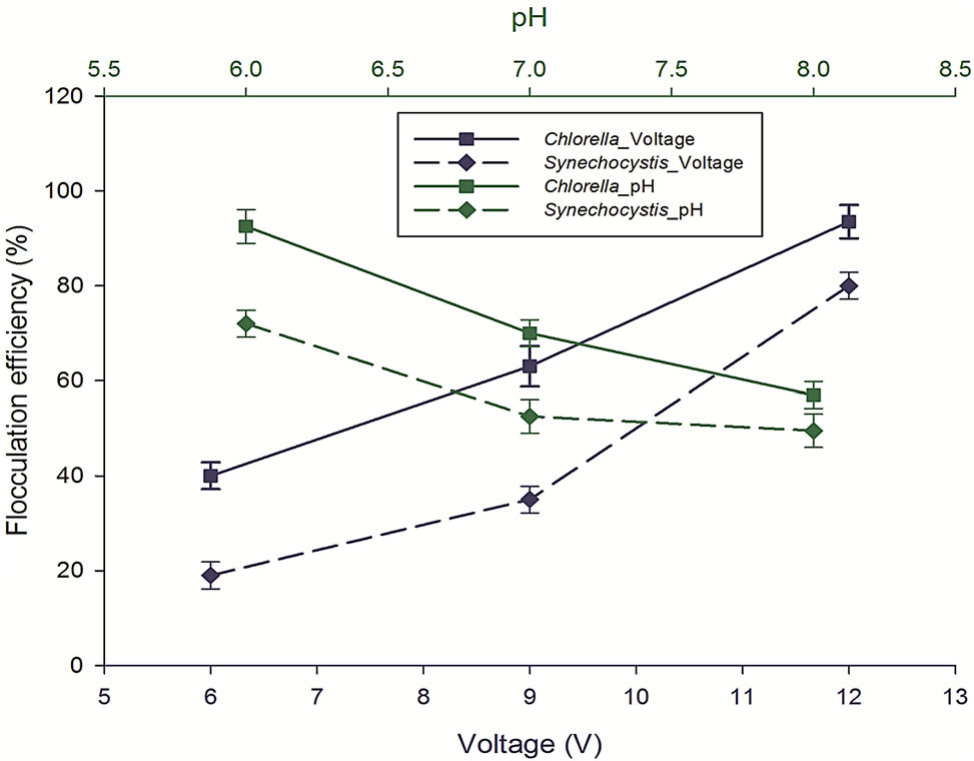
Effect of voltage and pH on the flocculation efficiency of *Chlorella* sp. MJ 11/11 and *Synechocystis* PCC 6803

With increasing voltage, flocculation efficiency was found to increase (Fig. 4). In the present study, three different voltage 6 V (5.967774 A/m^2^), 9 V (8.594347 A/m^2^), 12 V (10.9529 A/m^2^) of DC were applied. Maximum flocculation efficiency of 96 % was found at 12 V as compared to 42 and 66 % at 6 and 9 V, respectively, using *Chlorella* sp. MJ 11/11. But in case of *Synechocystis* PCC 6803 grown in BG-11 no coagulation-floatation was observed during 75 min of the reaction period. It might be due to lack of enough electrolyte concentration to facilitate the electro coagulation-floatation understudied range of voltage. With increasing the voltage, current density increases which determine the coagulant dosage rate, the bubble production rate, size and the flocs growth resulting faster removal efficiency at a higher voltage (Khosla et al. 1991). In this process, floatation of biomass was observed which is advantageous over sedimentation because it can be skimmed off easily which is favored in mass cultivation of algae (Edzwald 1993). Electrolysis of water produces hydrogen and oxygen gas at the electrodes. The bubbles produced at the anode (oxygen) and cathode (hydrogen) rise to the surface taking with them algal aggregates or flocs. The electrolysis leads to the flocculation and flotation of the algae at the same time without the usual addition of chemical flocculants.

When NaCl was added then a very fast reaction of coagulation-flocculation was observed in both type of microalgal suspension. Both organisms showed more than 90 % flocculation efficiency. Time was reduced from 30 to 15 min when the concentration of NaCl was increased from 0.5 g/L (13.73941 A/m^2^) to 1 g/L (16.62971 A/m^2^) (Table 3). The addition of NaCl increased the conductivity of suspension because of increased electrolyte concentration. Hence, high flocculation efficiency was observed in lesser time. When initial pH was kept in acidic condition, flocculation efficiency was found more than the alkaline condition in the same period (Fig. 4). In alkaline conditions, monomeric-hydroxy ferric anions are dominated in solution, which led to negative charges of the ferric hydroxide precipitates. In acidic and neutral pH range (6–7), ferric hydroxide precipitates and monomeric-hydroxo and polymeric ferric cations species are the primary species in the solution (Dermentzis et al. 2011). These positively charged precipitates would be easily adsorbed onto the negatively charged microalgae, which facilitated the removal of algae. Hence, Initial acidic pH was found more effective.

**Table 3:** Effect of NaCl on electro-flocculation (ECF) using *Chlorella* sp. MJ 11/11 and *Synechocystis* PCC 6803 (ECF was conducted at 12 V, initial pH 6.2, biomass concentration 1.5 g/L, electrode stainless steel)

NaCl concentration (g/L)	Flocculation efficiency (%)		Time (min)
	*Chlorella* sp. MJ 11/11	*Synechocystis* PCC 6803	
0	96.23 ± 1.6	0	75
0.5	96.03 ± 2.4	95.18 ± 1.3	30
1	95.08 ± 1.1	94.11 ± 2.2	15

As *Synechocystis* PCC 6803 is smaller in size. It has been reported that algae settle in the water column affected by intrinsic features such as buoyancy, surface charge, size, and shape of the cell. The cyanobacterial cells can regulate buoyancy with physical and chemical factors (Fernández and Ballesteros 2013; Chorus and Bartram 1999). Therefore, *Synechocystis* PCC 6803 sedimentation rate was observed to be lower than C*hlorella* sp. MJ 11/11. Flocculation efficiencies of different flocculation methods using both the organisms are shown in Table 4.

**Table 4:** Comparative study of different flocculation techniques for harvesting of microalgae

Methods	Concentration of flocculant (mg L^−1^)	Optimum pH	Settling time (min)	Maximum flocculation efficiency	
				*Chlorella* sp. MJ 11/11	*Synechocystis* PCC 6803
Chemical flocculation (Alum)	500	4.8–5.5	5–6	98.6 %	92.3 %
Chemical flocculation (ferric chloride)	400	6.2	5–6	98.6 %	91 %
Bio-flocculant/bio-polymer (Chitosan)	25	7.4	5–6	95–98 %	98 %
Auto-flocculation (at high pH)	NaOH	11.0–12.5	4	85 %	0–5 %
Electro-flocculation (Stainless-steel electrode)	12 V DC, NaCl (1 g/L)^a^	6.0–7.0	15	95–98 %	96 %

^a^Electro flocculation-coagulation with NaCl addition

### Cost estimation of flocculation

Cost estimation is one of the important factors associated with microalgal flocculation to make the process economically feasible. Other researchers have estimated the price of different flocculant mediated cell separation and have reported $0.7, 0.3, 31.1 for ferric chloride, potassium aluminum sulfate, and chitosan, respectively, for 1000 L of suspension culture of different microalgae (Rakesh et al. 2014). However, the concentration of biomass was not reported in their study. In the present study, cost of flocculants were found to be INR 210.00, 160.00, 1066.00, 79.80 for potassium aluminum sulfate, ferric chloride, chitosan, and electro-flocculation, respectively, for the recovery of 1 kg of algal biomass at the laboratory scale (Table 5). Thus, electro-flocculation was found a low-cost technique (INR 79.80 per kg dry biomass) than inorganic and biopolymer flocculants.

**Table 5:** Comparative cost economics of flocculation

	Alum	Ferric chloride	Chitosan	Electro-flocculation^a^
Cell concentration (g/L)	1	1	1	0.98
Flocculant quantity needed (mg/L)	500	400	10	–
Unit price of flocculant^b^ (Rs/kg)	420	400	106,600	–
Flocculation cost for 1 kg algal biomass (Rs/kg)^c^	210	160	1066	79.80

^a^Cost- power consumption in the process of electro-flocculation according to García-Pérez et al. (2014) and electricity price Rs 7.22/kWh (West Bengal, India)

^b^Price may vary supplier to supplier alum, ferric chloride (SRL), Chitosan (Sigma–aldrich)

^c^Cost involved in flocculation at a concentration, where a flocculation efficiency of ≥90 % was achieved

## Conclusions

In the present study, biomass production and suitable harvesting method were identified to recover algal biomass from the culture medium. In the air-lift reactor, high biomass productivity of C*hlorella* sp. MJ 11/11 was obtained which could be a potential feedstock for lipid production. Auto-flocculation process was found strain specific and effective at lower concentrations of biomass as compared to other flocculation processes. Potassium aluminum sulfate, FeCl_3_ showed potency in flocculation, but contamination may be an issue. On the other hand, chitosan was found eco-friendly but its high cost makes it unattractive for low-value products. Stainless-steel mediated electro-flocculation was found most suitable for the recovery of algal cells mainly due to low-cost, high flocculation efficiency, easy control of the process, floatation of biomass. However, further improvement in electro-flocculation can be done by optimizing different parameters of the process to make it more economically viable.

## References

[CR1] Barbosa MJ, Zijffers JW, Nisworo A, Vaes W, van Schoonhoven J, Wijffels RH (2005). Optimization of biomass, vitamins, and carotenoid yield on light energy in a flat-panel reactor using the A-stat technique. Biotechnol Bioeng.

[CR2] Chen CY, Yeh KL, Aisyah R, Lee DJ, Chang JS (2011). Cultivation, photobioreactor design and harvesting of microalgae for biodiesel production: a critical review. Bioresour Technol.

[CR3] Chorus EI, Bartram J (1999). Toxic cyanobacteria in water: a guide to their public health consequences, monitoring and management.

[CR4] Dermentzis K, Christoforidis A, Valsamidou E, Lazaridou A, Kokkinos N (2011). Removal of hexavalent chromium from electroplating wastewater by electro-coagulation with iron electrodes. Glob NEST J.

[CR5] Divakaran R, Pillai VNS (2002). Flocculation of algae using chitosan. J Appl Phycol.

[CR6] Duan J, Gregory J (2003). Coagulation by hydrolysing metal salts. Adv Colloid Interface Sci.

[CR7] Edzwald JK (1993). Algae, bubbles, coagulants, and dissolved air flotation. Water Sci Technol.

[CR8] Fernández CG, Ballesteros M (2013). Microalgae auto-flocculation: an alternative to high-energy consuming harvesting methods. J Appl Phycol.

[CR9] García-Pérez JS, Beuckels A, Vandamme D, Depraetere O, Foubert I, Parra R, Muylaert K (2014). Influence of magnesium concentration, biomass concentration and pH on flocculation of *Chlorella vulgaris*. Algal Research.

[CR10] Ghernaout D, Ghernaout B (2012). On the concept of the future drinking water treatment plant: algae harvesting from the algal biomass for biodiesel production—a review. Desalination Water Treat.

[CR11] Gouveia L, Oliveira AC (2009). Microalgae as a raw material for biofuels production. J Ind Microbiol Biotechnol.

[CR12] Grima EM, Belarbi EH, Fernández FA, Medina AR, Chisti Y (2003). Recovery of microalgal biomass and metabolites: process options and economics. Biotechnol Adv.

[CR13] Henderson R, Parsons SA, Jefferson B (2008). The impact of algal properties and pre-oxidation on solid-liquid separation of algae. Water Res.

[CR14] Khosla NK, Venkatachalam S, Somasundaran P (1991). Pulsed electrogeneration of bubbles for electroflotation. J Appl Electrochem.

[CR15] Mandal S, Mallick N (2009). Microalga *Scenedesmus obliquus* as a potential source for biodiesel production. Appl Microbiol Biotechnol.

[CR16] Milledge JJ, Heaven S (2013). A review of the harvesting of micro-algae for biofuel production. Rev Environ Sci Biotechnol.

[CR17] Nayak BK, Pandit S, Das D, Veiga MC, Kennes C (2013). Biohydrogen. Air Pollution Preventive Control.

[CR18] Nishifi L, Madronal GS, Guilhennez ALF, Vieira AM (2011). Cyanobacteria removal by coagulation/flocculation with seeds of the natural coagulant *Moringa oleifera* Lam. Chem Eng.

[CR19] Rakesh S, Saxena S, Dhar DW, Prasanna R, Saxena AK (2014). Comparative evaluation of inorganic and organic amendments for their flocculation efficiency of selected microalgae. J Appl Phycol.

[CR20] Roussy J, Vooren MV, Guibal E (2005). Chitosan for the coagulation and flocculation of mineral colloids. J Dispers Sci Technol.

[CR21] Scragg AH, Illman AM, Carden A, Shales SW (2002). Growth of microalgae with increased calorific values in a tubular bioreactor. Biomass Bioenergy.

[CR22] Skjanes K, Lindblad P, Muller J (2007). BioCO_2_—A multidisciplinary, biological approach using solar energy to capture CO_2_ while producing H_2_ and high value products. Biomol Eng.

[CR23] Sukenik A, Shelef G (1984). Algal autoflocculation–verification and proposed mechanism. Biotechnol Bioeng.

[CR24] Uduman N, Qi Y, Danquah MK, Forde GM, Hoadley A (2010). Dewatering of microalgal cultures: a major bottleneck to algae-based fuels. J Renew Sustain Energy.

[CR25] Vandamme D, Pontes SCV, Goiris K, Foubert I, Pinoy LJJ, Muylaert K (2011). Evaluation of electro-coagulation–flocculation for harvesting marine and freshwater microalgae. Biotechnol Bioeng.

[CR26] Vandamme D, Foubert I, Muylaert K (2013). Flocculation as a low-cost method for harvesting microalgae for bulk biomass production. Trends Biotechnol.

[CR27] Vlaski A, Breemen AN, Alaerts GJ (1997). The role of particle size and density in dissolved air flotation and sedimentation. Water Sci Technol.

[CR28] Wu Z, Zhu Y, Huang W, Zhang C, Li T, Zhang Y (2012). Evaluation of flocculation induced by pH increase for harvesting microalgae and reuse of flocculated medium. Bioresour Technol.

[CR29] Wyatt NB, Gloe LM, Brady PV, Hewson JC, Grillet AM, Hankins MG, Pohl PI (2012). Critical conditions for ferric chloride-induced flocculation of freshwater algae. Biotechnol Bioeng.

[CR30] Xu Y, Purton S, Baganz F (2013). Chitosan flocculation to aid the harvesting of the microalga *Chlorella sorokiniana*. Bioresour Technol.

[CR31] Yahi H, Elmaleh S, Coma J (1994). Algal flocculation-sedimentation by pH increase in a continuous reactor. Water Sci Technol.

[CR32] Yoo C, Jun SY, Lee JY, Ahn CY, Oh HM (2010). Selection of microalgae for lipid production under high levels carbon dioxide. Bioresour Technol.

